# Contemporary, postpandemic description of UK occupational therapy and physiotherapy practice to rehabilitate the upper limb after stroke: the SUPPLES 2 online survey

**DOI:** 10.1136/bmjopen-2024-095290

**Published:** 2025-09-21

**Authors:** Kathryn A Jarvis, Louise Connell, Rosemary Peel, Rachel C Stockley

**Affiliations:** 1 Stroke Research Team, School of Nursing and Midwifery, University of Lancashire, Preston, UK; 2 Lancaster Medical School, Lancaster University, Lancaster, UK; 3 University Hospitals of Morecambe Bay NHS Foundation Trust, Kendal, Cumbria, UK

**Keywords:** Stroke, Surveys and Questionnaires, REHABILITATION MEDICINE

## Abstract

**Abstract:**

**Objectives:**

To provide a contemporary, postpandemic description of UK occupational therapy and physiotherapy practice to rehabilitate the upper limb after stroke.

**Setting:**

A national online survey, first undertaken in 2018 (prepandemic), was readministered to describe postpandemic practice.

**Participants:**

The survey was distributed to UK-based occupational therapists and physiotherapists working with people after stroke, via professional and social networks.

**Primary measures:**

Shaped by the Template for Intervention Description and Replication Checklist, the survey collected and subsequently analysed the content, frequency and duration of upper limb rehabilitation after stroke.

**Results:**

A total of 122 occupational therapists (n=42) and physiotherapists (n=80) currently working clinically, across in-patient, out-patient and community settings, in the UK completed the survey. Respondents reported treating the upper limb a median of three times a week (IQR 2–4; range 0–6) for a median of 25 min (IQR: 20–35; range 3–60; n=119). Repetitive, functionally-based activities were the most commonly reported interventions for mild (n=93; 81%) and moderate (n=72; 64%) impairment. Stretching (n=73; 66%) and positioning (n=49; 45%) were most frequently reported for severe impairment. In each of the three impairment categories, a larger number of interventions were reported than in the 2018 survey.

**Conclusions:**

While the pandemic promoted the use of virtual interventions, most therapists had returned to face-to-face interventions. The findings highlight that the current reported provision of upper limb therapy continues to be markedly less than the dose shown to be effective. The study provides important data which can be used to judge the success of attempts to align practice with new guidelines and inform ‘usual therapy’ for the upper limb after stroke in comparative studies.

**STRENGTHS AND LIMITATIONS OF THIS STUDY**

- Repeating a detailed cross-sectional survey facilitated an in-depth analysis of postpandemic and a comparison with prepandemic, upper limb therapeutic practice following stroke.
- The detailed survey design enabled the collection of key details about the content, frequency and duration of therapeutic practice for differing arm impairment severity after stroke.
- Recruitment may have been impacted by the length of the survey.
- Findings of the survey are limited by its reliance on self-report.

## Background

 Diminished upper limb (arm and hand) function is a frequent consequence of stroke, with estimations that between 40% and 70% of stroke survivors will experience impairment of their upper limb.^1^ This can lead to significant disability and pose challenges when attempting basic everyday tasks, limit independence and quality of life.^2^ Dependent on the severity and presentation of the impairment, global (including UK) guidance recommends the use of evidence-based therapeutic interventions that include repetitive task training, constraint-induced movement therapy, strength training, mirror therapy, mental practice, functional electrical stimulation, sensory-specific training and education in the care of the upper limb.^3^,^5^ For stroke survivors with motor recovery goals, the current UK guidelines^3^ recommend a minimum of 3 hours of multidisciplinary therapy a day, for at least 5 days a week.

In 2018, we conducted a national UK cross-sectional online survey. This Survey of Upper Limb Therapy after Stroke (SUPPLES-UK) was developed using UK stroke guidelines and previous studies investigating upper limb recovery following stroke and described stroke therapy for the upper limb.^6^ SUPPLES-UK was completed by 156 occupational therapists or physiotherapists working clinically with stroke survivors and provided a snapshot of the commonly used treatments of the upper limb in the UK. The findings showed that, in 2018, although some of the evidence-based treatments (eg, repetitive task training and constraint-induced movement therapy) were being used widely in clinical practice, others, such as mental practice and functional electrical stimulation, were not.^6^

Since SUPPLES-UK was conducted, a global pandemic disrupted healthcare, including stroke recovery and rehabilitation. In the UK, this led to a reduction in occupational therapy and physiotherapy treatment time,^7^ with approximately 40% of stroke survivors reporting that they did not receive enough rehabilitation, and over 50% of stroke survivors reporting cancelled or postponed appointments during the pandemic.^7^ Alongside the reduction in therapy time, aspects of therapy delivery were untenable or needed to be modified significantly.^8^ These challenges necessitated new and flexible ways of working through innovative methods of service delivery. Some of these involved reconfigurations of existing resources while others involved adoption of technology and new ways of working.^7^,^9^ Examples of these changes included a greater reliance on virtual interventions and support groups, development of interdisciplinary approaches and supported self-management.^8 9^

In the wake of the pandemic, it is not known if these enforced changes, along with other challenges, for example, difficulties reclaiming rehabilitation space that had been reassigned during the pandemic,^10^ have impacted therapy provision in stroke rehabilitation. The policy context in this timeframe has also changed, with the National Health Service publishing a new model for integrated community stroke services.^11^ This approach has changed the rehabilitation landscape, as the model seeks to provide early and effective community stroke rehabilitation and disability management to stroke survivors leaving hospital.

The aim of this study is, therefore, to provide a contemporary, postpandemic description of occupational therapy and physiotherapy practice to rehabilitate the upper limb after stroke and to highlight changes that have occurred since the original SUPPLES-UK survey in 2018. There was no a priori hypothesis.

## Methods

### Data collection tool

The original cross-sectional online SUPPLES-UK^6^ was revised by two occupational therapists (RP and KAJ) and two physiotherapists (RCS and LC). The revisions ensured that SUPPLES 2-UK reflected changes to the evidence base, practice following the COVID-19 pandemic, and the increased availability of digital technology solutions on the market. Additional questions sought information about: (1) the format of intervention delivery (ie, estimation of percentage of time spent in face-to-face, virtual-telephone and virtual-video calls); (2) the use of technology-based interventions (ie, frequency of use of apps, electrical stimulation, virtual reality and wearable technology); (3) the use of sensory interventions and (4) whether respondents had previously completed the 2018 survey. Wording of questions was clarified where there had been inconsistencies in interpretation of the question in the 2018 survey responses. For example, the question requesting the postcode of the respondent’s primary place of work was infrequently completed in the original survey. In SUPPLES 2-UK, the survey requested the country in which the respondent worked.

The final SUPPLES 2-UK survey comprised 57 closed, Likert and free-text items (detail provided in online supplemental file 1). The original and revised survey design was guided by the Template for Intervention Description and Replication (TIDieR),^12^ with questions designed to collect data about the location (where), content (what), frequency and duration (how much) of reported therapy. Information about the respondents (who) was also collected. To reduce bias arising from estimating time, respondents were asked to estimate a percentage where this was possible (eg, the percentage of clinical time spent working with people who have had a stroke). One question asked for an estimation of the number of minutes typically spent directly undertaking upper limb treatment with a person who has upper limb deficits after stroke.

The National Institute of Health Stroke Scale definitions of upper limb impairment^13^ were used in seven questions to gain further detail (0–1 mild—able to lift and hold arm up against gravity for 10 s; 2 moderate—some effort against gravity, but the arm cannot get to or maintain the proper position and drifts before 10 s; 3–4 severe—unable to move against gravity or no voluntary movement).

The original survey SUPPLES-UK was piloted by three therapists, and the second version SUPPLES 2-UK was piloted by an additional two therapists, and the final survey was refined based on this feedback.

The survey was designed and distributed using the Online Surveys tool (www.onlinesurveys.ac.uk). The final survey is available in supplementary material (online supplemental file 1).

### Recruitment

The recruitment strategy aimed to reach occupational therapists and physiotherapists working with people living with stroke and used multiple channels to maximise this reach. The survey was distributed through professional channels, using email distribution (The Association of Chartered Physiotherapists Interested in Neurology, where an initial email was followed by a second email approximately 6 weeks later), professional publications (OT News, the Royal College of Occupational Therapy-Specialist Section Neurological Practice Newsletter) and social media (X). The team also shared the information through their networks and at conferences. The complex nature of recruitment using networking capital to gain a maximum response prevented an accurate estimate of the number of recruitment invitations distributed and response rate. Open from 9 November 2022 until 31 January 2023, the survey was available for 12 weeks.

Therapists following the online link could access an information sheet (online). This provided information about the purpose of the study, the steps involved in participating, the reason for the invitation and the option to participate (or not). Consent was implied when a therapist completed the survey. Respondents confirmed they were an occupational therapist or physiotherapist and that they were currently working with stroke survivors before gaining access to the full survey. All responses were anonymous.

### Data cleaning

Data were reviewed and cleaned by one researcher (KAJ). If a respondent reported time since qualification or time working with people following stroke as less than a year, this was classified as 1 year if no additional detail was given. Similarly, if respondents reported spending <5% of time on an activity, this was recorded as five unless other information was available to increase accuracy.

Where respondents reported working in more than one location (eg, in both community and inpatient settings), the location in which they spent 75% or more of their time was used. Location data were included only if a respondent worked 75% of their time in one location. Where respondents reported a range in an answer in the survey, for example, 40–60 min, the median value was used. Where a question asked for a percentage of time, and this was reported by the respondent as a number of days, the data were converted to a percentage, based on the assumption of five working days in a week.

### Data analysis

Consistent with the aims of this study, demographic details, treatment content, frequencies and durations were summarised using descriptive statistics. The data were examined for normality by visual inspection of distribution; data that were not normally distributed were reported as medians, IQRs and ranges. All available data for each survey item were included, with missing data denoted by a reduced sample (n <122).

Free-text answers about individual interventions were initially coded by one researcher (KAJ) and independently verified by another (RP). Any disagreements in coding were resolved by a third person (RCS). These codes were reviewed by the full research team and grouped based on their features and rehabilitation objective (code list available in online supplemental file 2). Where interventions were reported by <10% of respondents, we made the assumption that these were rarely used by therapists and did not include these interventions in the final analysis.

The TIDieR framework structured the data analysis. This paper reports who provided treatments (who; physiotherapists, occupational therapists, others), where respondents were based (where), treatment content (what) and frequency and duration (when and how much). Analyses were undertaken using Microsoft Excel and SPSS V.28.

### Patient and public involvement

Patients and the public were not involved in this research.

## Findings

The 122 therapists who responded to SUPPLES 2-UK were included in the analysis.

### Who?

All respondents were providers of upper limb therapy poststroke. They comprised more physiotherapists (PT) than occupational therapists (OT) (80 physiotherapists, 66%; 42 occupational therapists, 34%). Eight respondents (7%) had completed the previous SUPPLES-UK survey in 2018, and 85 respondents (70%) had not, with the other 29 respondents unsure (28: 23%) or not responding (1: 1%). The majority of respondents reported an undergraduate degree as their highest qualification (n=77; 63%), 30 held a master’s degree (25%), 5 held a PhD (4%) and 4 a vocational diploma (3%). Five had completed some master’s modules and/or had some postgraduate (PG) qualifications (PG certificate or similar; 4%) and one participant did not respond. Respondents were a median of 15 years since qualification (range 1–33; IQR: 8–22; n=122). Respondents had worked with people after stroke for a median of 10 years (range 0.25–30, IQR: 4–15; n=122), and they reported spending a median of 80% of their clinical time working with people after stroke (range: 8%–100%; IQR: 50%–100%; n=121).

Respondents (115/121) identified rehabilitation assistants (n=21), family/carer/friend (n=83), other members of the multidisciplinary team (n=6) as other providers of therapy for the upper limb.

### Where?

**Figure 1:**
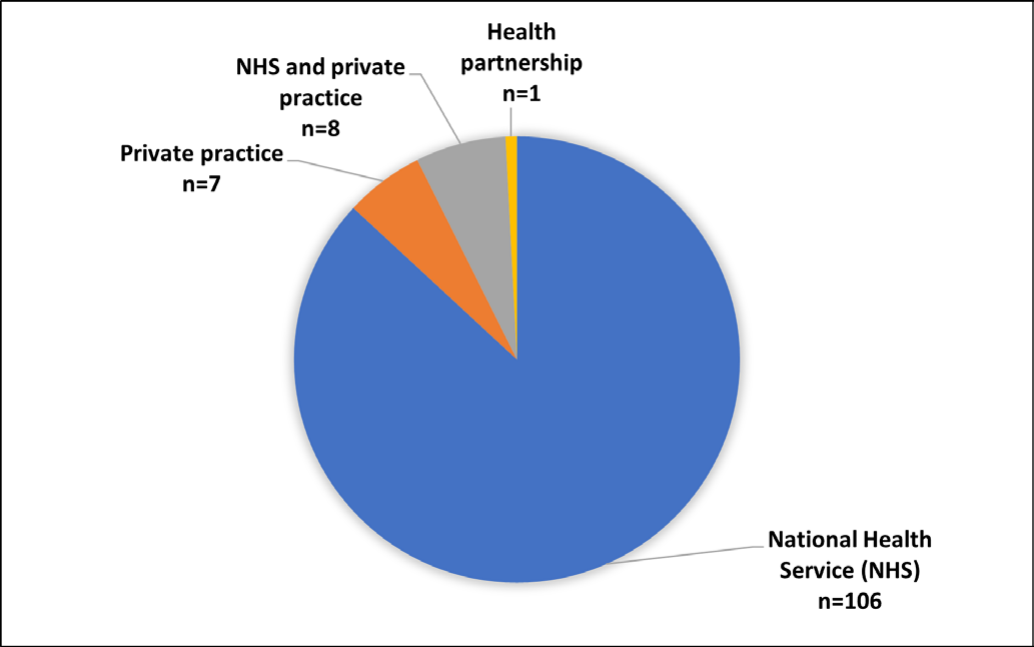
Therapist employment organisations.

The employment organisation for the 122 responding therapists is shown in figure 1. 79 respondents (65%) reported working in only one practice area (inpatient: n=38 (48%); outpatient: n=4 (5%); community: n=37 (47%)). Of those that reported spending over 75% of their time in a single setting (n=101), 46 (46%) worked in inpatient rehabilitation, 5 (5%) in outpatient rehabilitation and 50 (50%) in community rehabilitation. One respondent (1%) recorded that they worked in an ‘other’ rehabilitation setting which was not specified. The remainder (n=18) did not spend more than 75% of their time in a single setting or did not respond (n=3).

Respondents indicating the nation in which they were based (n=120) showed respondents from England (101/122: 83%), Scotland (13/122: 11%) and Wales (6/122: 5%). There were no respondents from Northern Ireland.

### What?

#### Method of delivery

Rehabilitation was predominantly being delivered face-to-face. The median percentage face-to-face intervention time was 100% (IQR 90–100; range 50–100). Although the median percentage of time for virtual interventions using telephone was 0% (IQR 0–7.5; range 0–33) and using video calls was 0% (IQR 0–0; range 0–30), some respondents did report using virtual interventions. These were most evident in the community setting, where, of the 71 respondents who reported undertaking some work in the community, 21 (30%) reported using video calls and 32 (45%) telephone calls.

#### Content

**Figure 2:**
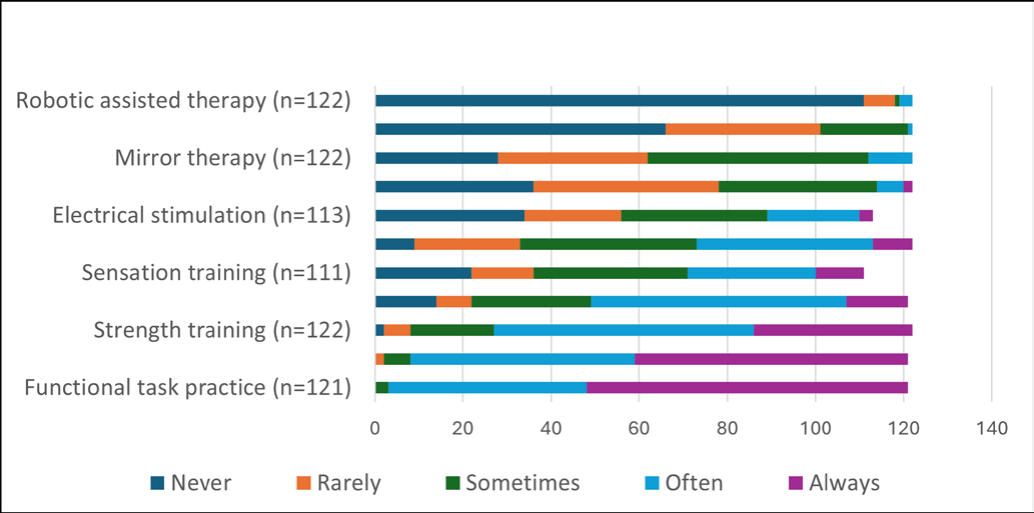
Summary of frequency of use for individual interventions.

Therapists were asked how often they used a range of individual interventions. The responses are summarised in figure 2 (further detail provided in online supplemental file 3).

Across the 11 types of interventions, a reason for ‘never’ using an intervention was reported 327 times. The most frequent reason was that the respondent did not have access to the intervention (211/327; 65%), with robotics and video/gaming/virtual reality accounting for 165/327 (50%). Not having received training was also a factor (82/327; 25%), with constraint-induced movement therapy and sensation training accounting for 33/327 (10%).

#### Mild deficits

Respondents (n=121) reported spending a median of 30% (IQR: 20–50; range: 5–100) of a typical therapy session on treating the upper limb where mild deficits were present. 28 interventions were listed by 115 respondents, with seven interventions reported by more than 10% of the respondents (table 1).

**Table 1:** Interventions for mild upper limb deficits reported by more than 10% of the respondents

Intervention	N=	%
Functional repetitive practice	93	81
Strengthening/exercise	60	53
Graded Repetitive Arm Supplementary Program	32	28
Constraint induced movement therapy	24	21
Dexterity	18	16
Sensory re-education	17	15
Stretches/range of motion	14	12

Shading indicates interventions reported by ≥10% of respondents in the 2023 survey but not in the 2018 survey. No additional interventions were reported by ≥10% in the 2018 survey.

#### Moderate deficits

Respondents (n=121) reported spending a median of 50% (IQR: 30–50; range:10–100) of a session on upper limb interventions for those with moderate upper limb deficits. 31 interventions were listed by 113 respondents; 11 interventions were reported by more than 10% of the respondents (table 2).

**Table 2:** Interventions for moderate upper limb deficits reported by more than 10% of the respondents

Intervention	N=	%
Functional repetitive practice	72	64
Strengthening/exercise	59	52
Electrical stimulation	38	34
Specialist equipment	28	25
Graded Repetitive Arm Supplementary Program	26	23
Stretches/range of motion	25	22
Sensory re-education	25	22
Positioning	18	16
Mirror therapy	15	13
Facilitation	14	12
Constraint-induced movement therapy	12	11

Shading indicates interventions reported by ≥10% of respondents in the 2023 survey but not in the 2018 survey. No additional interventions were reported by ≥10% in the 2018 survey.

Specialist equipment refers to technologies that have been specifically designed to increase the function of the upper limb (eg, SaeboGlove, Gripable, Biometrics).

#### Severe deficits

Where a severe upper limb deficit was present, respondents (n=121) reported spending a median of 30% (IQR: 20–50; range: 0–100) of a session providing upper limb interventions. Of the 31 interventions listed by 110 respondents, nine interventions were reported by more than 10% of the respondents (table 3).

**Table 3:** Interventions for severe upper limb deficits reported by more than 10% of the respondents

Intervention	N=	%
Stretches/range of motion	73	66
Positioning	49	45
Sensory re-education	40	36
Electrical stimulation	36	33
Splints and orthoses	32	29
Strength/exercises	26	24
Mirror box	22	20
Mental practice	16	15
Weight-bearing	11	10

Shading indicates interventions reported by ≥10% of respondents in the 2023 survey but not in the 2018 survey. No additional interventions were reported by ≥10% in the 2018 survey.

#### Unsupervised activities

Of the respondents (n=121), 114 (94%) agreed that they would routinely ask people with mild arm deficits to undertake unsupervised activities for their upper limb in addition to therapist-led treatment. This decreased to 113 (93%) of respondents agreeing for people with moderate arm deficits and 99 (81%) for people with severe arm deficits. The unsupervised activities reported by 10% or more respondents are summarised in table 4.

**Table 4:** A summary of the activities that therapists routinely asked stroke survivors to undertake outside of therapy

Intervention	Mild n (%)	Moderate n (%)	Severe n (%)
Repetitive task training (including functional tasks)	72 (63%)	50 (44%)	
Strength/exercises	40 (35%)	50 (44%)	
Graded Repetitive Arm Supplementary Program	26 (23%)	22 (19%)	
Dexterity	18 (16%)		
Stretches/range of motion		22 (19%)	45 (45%)
Sensory re-education		15 (13%)	22 (22%)
Electrical stimulation		12 (11%)	
Mental practice			15 (15%)
Splinting			13 (13%)
Education			11 (11%)

### How much?

#### Frequency

Respondents (n=117) reported that occupational therapists and physiotherapists provided treatment for the upper limb a median of three times a week (IQR 2–4; range 0–6). The physiotherapists (n=79) mirrored these findings (median 3; IQR 2–4; range 0–6). Occupational therapists (n=38) reported providing treatment for the upper limb less frequently (median 2; IQR 1–2.5; range 1–6).

**Figure 3:**
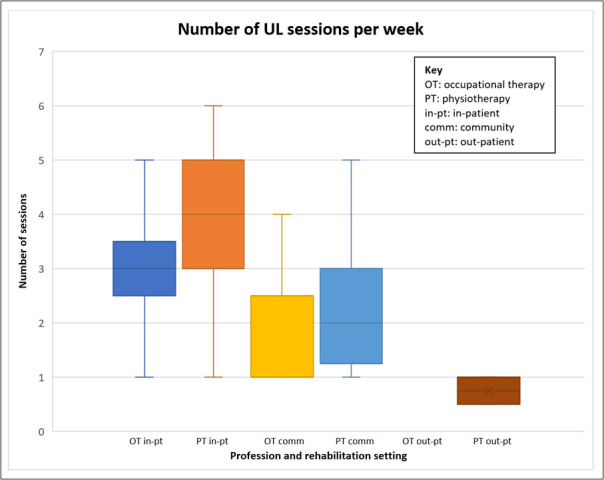
Number of upper limb (UL) therapy sessions per week (setting and profession).

The frequency of treatment sessions per week varied depending on setting. This is shown by profession in figure 3. Overall, based on the respondents who spent at least 75% in one setting (n=101), patients in inpatient settings received more frequent treatment (median 4; IQR 3–5, range 1–6; n=46) than those in general community (median 1; IQR 0.5–1, range 0.5–1; n=5) and outpatient settings (median 2; IQR 1–3, range 1–5; n=48 (2 non-responders)).

#### Duration

Respondents estimated typically spending a median of 25 min (IQR: 20–35; range 3–60; n=119) within each therapy session directly engaged in upper limb treatments (‘time on task’). Data from the 98 respondents who reported spending over 75% of their clinical time in one clinical setting indicated that there was variation between the clinical settings: inpatient n=44, median 20 min (IQR: 15–30 min); community n=49, median 30 min (IQR: 20–45 min); outpatient n=5, median 30 min (IQR: 17.5–46.25 min).

Overall, this equates to an individual therapist in 2023 offering an estimated 25 min of upper-limb focused therapy, three times a week.

## Discussion

This research aimed to provide a contemporary, postpandemic description of UK occupational therapy and physiotherapy practice (content, frequency and duration) to rehabilitate the upper limb after stroke. We found that respondents were treating the upper limb a median of three times a week (IQR 2–4; range 0–6) for a median of 25 min (IQR: 20–35; range 3–60; n=119). These findings appear to reflect the findings from 2018 (28 min, three times a week). However, in each of the three impairment categories (mild, moderate and severe), a greater number of interventions were reported in 2023 than in 2018, with interventions reported by 10% or more therapists increasing for mild (from 4 to 7), moderate (from 5 to 11) and severe (from 3 to 9) impairment. This seems to indicate a decreased consensus around which interventions should be used for each impairment category, particularly in the severe impairment category where there were six additional interventions being reported. While this could be explained by therapists using the range of recommended interventions from UK guidelines,^3^ this survey indicated that specific interventions for the most severe upper limb deficits, such as splinting and weight bearing, were being used despite not being recommended for routine practice.

The delivery of rehabilitation remotely,^14^ including virtual consultations, assessments and therapeutic interventions,^15^ was established and became a bedrock for therapists during the COVID-19 pandemic.^15^ While research has shown that the pandemic has increased rehabilitation professionals’ willingness to use technology and their digital skills,^16^ our survey indicates that, despite a strategic enthusiasm for virtual consultations,^17 18^ therapists have almost completely returned to face-to-face delivery. It is unclear if this is because therapists feel that face-to-face results in a more effective response, is more acceptable or whether implementation challenges have prevented scale-up and routinising of this approach.^19^

Some demarcation was evident in the interventions reported across the three impairment categories. Repetitive, functionally based activities were the most commonly reported intervention for mild and moderate impairment. However, moderate impairment interventions also included specialist equipment, such as electrical stimulation, and technologies, including Saebo gloves and Gripable. These interventions were not reported in the 2018 survey and may signal a move towards interventions that have the potential to increase the time engaged in therapeutic activity. Severe upper limb impairments were characterised by interventions to maintain available range of movement and increasing awareness of the upper limb, with electrical stimulation, sensory re-education, mental practice and mirror therapy^20^ offering potential routes to prime neural pathways.

The intensity of upper limb interventions remains markedly lower than that shown to have benefit.^21^ SUPPLES 2-UK indicates that respondents were recommending unsupervised therapy; when used judiciously, this has the potential to increase intensity but is unlikely to be suitable for all people after stroke (eg, those with significant cognitive deficits). Technology solutions may offer a route to augment face-to-face therapy outside of traditional therapy sessions.^22^ Yet, SUPPLES2-UK identifies that the use of technology in upper limb recovery remains an underdeveloped area, potentially limiting this advancement. Further work is required to ensure effective technology is readily available and accessible for those undertaking upper limb rehabilitation.

The findings from our study provide an important reflection of ‘usual therapy’ for the upper limb after stroke and so can be used to inform usual care interventions for comparative studies as well as guiding evaluation in studies seeking to examine alterations in practice. By describing therapy practice just prior to the introduction of the new UK stroke guideline, SUPPLES 2-UK provides an important baseline. A future SUPPLES study could enable a view of change over time, as healthcare organisations aim to meet the recommendations of the current stroke guideline.

### Implications for practice

The SUPPLES 2-UK survey indicates a similar intensity of therapy for the upper limb in 2023 compared with 2018 and underlines a continuing shortfall in the amount of therapy offered to people after stroke. Service provision needs to enable therapists to rise to the substantial challenge of delivering evidence-based interventions tailored to meet the needs of individual stroke survivors at the level required. This will require changes at every level in health systems and require investment. The current stroke guidance^3^ recommends a mixture of face-to-face and online approaches but does not offer guidance about the ratio of the approaches, nor does this advice relate specifically to upper limb interventions. If therapists are going to achieve the recommended level of intensity, they will need to be empowered to creatively find new ways of approaching their therapeutic offering. These may include using technologies,^23 24^ group-based^24 25^ and self-management interventions^26^ where these are supported by evidence of effectiveness. Managing change to services and implementing new approaches or systems takes considerable effort, time and skill.^27^ Therapists are being asked to embrace not only evidence-based practice but also tools and approaches to enable these new ways of working. Time to undertake this development work needs to be allocated and supported within the organisation if therapists are going to be able to meet the therapeutic intensity required for upper limb recovery.

### Limitations

Repeating the 2018 survey provided insights into postpandemic therapeutic practice. The use of the TIDieR checklist to underpin both the SUPPLES-UK and SUPPLES 2-UK ensured that the data collected gave a comprehensive picture of upper limb stroke rehabilitation in 2018 and 2023. However, the responses were self-reported, so it must be recognised that the findings reflect the therapist respondents’ perceptions of what they do, rather than the findings being based on observation. The study is potentially limited by selection bias, as respondents are more likely to be therapists who are more confident in their practice, adhere to evidence-based guidance and engage with online resources for their continued professional development. There were fewer responders to the 2023 survey (n=122) compared with the 2018 survey (n=156), despite using a similar recruitment approach. This included the wide promotion of the survey using a range of professional networks to maximise the sample. We recognise that increasing pressure on NHS staff^28^ was likely to reduce the time therapists had to engage in research,^29^ impacting survey responses. We aimed to mitigate these challenges by keeping the survey link open for a period of 12 weeks, streamlining the survey and advising potential respondents that the survey was likely to take 10–15 min to complete. However, in a busy workforce, the length of the survey may have impacted the number of therapists who felt they could take time to respond. While the response rate was relatively low, most of those that did complete the survey provided a full, or almost full, data set. The SUPPLES 2-UK sample reflected the SUPPLES-UK sample (highest qualification, type of employer, time since qualification and working with people living with stroke). SUPPLES 2-UK predominantly comprised experienced physiotherapy and occupational therapy participants working in a range of settings in three of the four UK nations, with the proportion of therapists from the individual UK nations reflecting the current workforce (England occupational therapy/physiotherapy 82%/81%; Scotland occupational therapy/physiotherapy 8%/7%; Wales occupational therapy/physiotherapy 5%/5%; Northern Ireland occupational therapy/physiotherapy 3%/3%).^30 31^ We are, therefore, confident that despite these limitations, the findings do reflect current practice.

## Conclusion

Frequency and duration of upper limb interventions postpandemic were similar to prepandemic levels. The intensity of therapy was lower than has been shown to positively influence motor recovery and did not meet the stroke guidance at the time of the survey, or the subsequent, updated version of the guidance. There are some changes in the treatments used by therapists following the pandemic. Most, but not all, were supported by national and international stroke guidelines. Considerable work is required to provide the context and systems to enable therapists to rise to the challenge to deliver evidence-based interventions at the level required. The SUPPLES 2-UK study provides a baseline of upper limb therapeutic interventions in the UK to inform future studies and guidelines.

## Supplementary material

10.1136/bmjopen-2024-095290 online supplemental file 1

10.1136/bmjopen-2024-095290 online supplemental file 2

10.1136/bmjopen-2024-095290 online supplemental file 3

## Data Availability

Data are available in a public, open access repository.
