# Supplementary material for: Contemporary, postpandemic description of UK occupational therapy and physiotherapy practice to rehabilitate the upper limb after stroke: the SUPPLES 2 online survey
Source: BMJ Open. 2025 Sep 21;15(9):e095290. doi: 10.1136/bmjopen-2024-095290 (PMC12458861; doi:10.1136/bmjopen-2024-095290)
Supplement: online supplemental file 1 [file bmjopen-15-9-s001.docx]

**SUPPLES 2-UK Questions**

1. Are you a Physio or Occupational Therapist working clinically in the UK? [closed]

2. How many years have you been qualified? [closed]

3. What is your highest academic qualification? [closed]

3.a. If you selected Other, please specify: [free text]

4. How many years have you worked with people who have had a stroke? [closed]

5. Do you currently work clinically with stroke survivors with upper limb deficits at any stage of their rehabilitation? [closed]

6. Where are you currently employed? Please tick all settings that apply. [closed]

6.a. If you selected Other, please specify: [free text]

7. Please tell us the region/country in which you work (e.g. Northern Ireland/England/Scotland/Wales/Other) and, if applicable, please tell us which Integrated Stroke Delivery Network you are in. [closed]

8. On average, what percentage of your clinical time is spent working with people who have had a stroke? [closed]

9. Please estimate when working with people after stroke what percentage of this time is spent in face to face or virtual rehabilitation? [closed]

9.1.a. Face to face - Percentage of time using this approach to deliver rehabilitation for people after stroke [closed]

9.2.a. Virtual - telephone - Percentage of time using this approach to deliver rehabilitation for people after stroke [closed]

9.3.a. Virtual - video calls - Percentage of time using this approach to deliver rehabilitation for people after stroke [closed]

9.4.a. Virtual - other - Percentage of time using this approach to deliver rehabilitation for people after stroke [closed]

9.5.a. Other - Percentage of time using this approach to deliver rehabilitation for people after stroke [closed]

10. Thinking about only about your role working with people after stroke, please provide: 1. an approximate percentage of the time you spend in each setting (e.g. 40% inpatient rehabilitation, 60% community rehabilitation) in the first column of the table. This column should add up to 100%. For each setting in which you work, please use the subsequent columns to provide: 2. an estimate of the percentage of your time, when working directly with people after stroke, that is conducted face to face or virtually in each setting (e.g. 60% face to face, 30% using video and 10% using telephone).

10.1.a. Inpatient rehabilitation - Percentage of your time spent in each area (this should represent just the time you work with people after stroke, and, therefore, this column should add up to 100%) [closed]

10.1.b. Inpatient rehabilitation - Percentage of time spent delivering rehabilitation face to face [closed]

10.1.c. Inpatient rehabilitation - Percentage of time using video to deliver rehabilitation [closed]

10.1.d. Inpatient rehabilitation - Percentage of time using telephone calls to deliver rehabilitation [closed]

10.2.a. Outpatient rehabilitation - Percentage of your time spent in each area (this should represent just the time you work with people after stroke, and, therefore, this column should add up to 100%) [closed]

10.2.b. Outpatient rehabilitation - Percentage of time spent delivering rehabilitation face to face [closed]

10.2.c. Outpatient rehabilitation - Percentage of time using video to deliver rehabilitation [closed]

10.2.d. Outpatient rehabilitation - Percentage of time using telephone calls to deliver rehabilitation [closed]

10.3.a. Community rehabilitation - Percentage of your time spent in each area (this should represent just the time you work with people after stroke, and, therefore, this column should add up to 100%) [closed]

10.3.b. Community rehabilitation - Percentage of time spent delivering rehabilitation face to face [closed]

10.3.c. Community rehabilitation - Percentage of time using video to deliver rehabilitation [closed]

10.3.d. Community rehabilitation - Percentage of time using telephone calls to deliver rehabilitation [closed]

10.4.a. Other - Percentage of your time spent in each area (this should represent just the time you work with people after stroke, and, therefore, this column should add up to 100%) [closed]

10.4.b. Other - Percentage of time spent delivering rehabilitation face to face [closed]

10.4.c. Other - Percentage of time using video to deliver rehabilitation [closed]

10.4.d. Other - Percentage of time using telephone calls to deliver rehabilitation [closed]

11. Please tell us about additional technology used to support upper limb rehabilitation for people who have had a stroke. This may be either within therapy sessions or in addition to supervised therapy. Please indicate how frequently you have used each form of technology.

11.1.a. Apps (e.g. providing stroke specific exercises for the upper limb - please tell us the name or about these apps in the last column) - Frequency of use [Likert]

11.1.b. Apps (e.g. providing stroke specific exercises for the upper limb - please tell us the name or about these apps in the last column) - Is this technology typically used in a supervised therapy session? [closed]

11.1.c. Apps (e.g. providing stroke specific exercises for the upper limb - please tell us the name or about these apps in the last column) - Is this technology typically used by the patient outside of supervised therapy? [closed]

11.1.d. Apps (e.g. providing stroke specific exercises for the upper limb - please tell us the name or about these apps in the last column) - Please tell us any further information you feel is relevant [free text]

11.2.a. Brain-computer interfaces - Frequency of use [Likert]

11.2.b. Brain-computer interfaces - Is this technology typically used in a supervised therapy session? [closed]

11.2.c. Brain-computer interfaces - Is this technology typically used by the patient outside of supervised therapy? [closed]

11.2.d. Brain-computer interfaces - Please tell us any further information you feel is relevant [free text]

11.3.a. Electrical Stimulation (e.g. TENS, SaeboStim) - Frequency of use [Likert]

11.3.b. Electrical Stimulation (e.g. TENS, SaeboStim) - Is this technology typically used in a supervised therapy session? [closed]

11.3.c. Electrical Stimulation (e.g. TENS, SaeboStim) - Is this technology typically used by the patient outside of supervised therapy? [closed]

11.3.d. Electrical Stimulation (e.g. TENS, SaeboStim) - Please tell us any further information you feel is relevant [free text]

11.4.a. Robotics (e.g. Armeo) - Frequency of use [Likert]

11.4.b. Robotics (e.g. Armeo) - Is this technology typically used in a supervised therapy session? [closed]

11.4.c. Robotics (e.g. Armeo) - Is this technology typically used by the patient outside of supervised therapy? [closed]

11.4.d. Robotics (e.g. Armeo) - Please tell us any further information you feel is relevant [free text]

11.5.a. Virutal reality gaming using commerical systems (e.g. Nintendo Wii, Xbox) - Frequency of use [Likert]

11.5.b. Virutal reality gaming using commerical systems (e.g. Nintendo Wii, Xbox) - Is this technology typically used in a supervised therapy session? [closed]

11.5.c. Virutal reality gaming using commerical systems (e.g. Nintendo Wii, Xbox) - Is this technology typically used by the patient outside of supervised therapy? [closed]

11.5.d. Virutal reality gaming using commerical systems (e.g. Nintendo Wii, Xbox) - Please tell us any further information you feel is relevant [free text]

11.6.a. Virtual reality gaming using rehabilitation-specific systems (e.g. NeuroBall, YouGrabber) - Frequency of use [Likert]

11.6.b. Virtual reality gaming using rehabilitation-specific systems (e.g. NeuroBall, YouGrabber) - Is this technology typically used in a supervised therapy session? [closed]

11.6.c. Virtual reality gaming using rehabilitation-specific systems (e.g. NeuroBall, YouGrabber) - Is this technology typically used by the patient outside of supervised therapy? [closed]

11.6.d. Virtual reality gaming using rehabilitation-specific systems (e.g. NeuroBall, YouGrabber) - Please tell us any further information you feel is relevant [free text]

11.7.a. Wearable technologies (e.g. actigraph, smart watches) - Frequency of use [Likert]

11.7.b. Wearable technologies (e.g. actigraph, smart watches) - Is this technology typically used in a supervised therapy session? [closed]

11.7.c. Wearable technologies (e.g. actigraph, smart watches) - Is this technology typically used by the patient outside of supervised therapy? [closed]

11.7.d. Wearable technologies (e.g. actigraph, smart watches) - Please tell us any further information you feel is relevant [free text]

11.8.a. Other - please specify in the last column - Frequency of use [Likert]

11.8.b. Other - please specify in the last column - Is this technology typically used in a supervised therapy session? [closed]

11.8.c. Other - please specify in the last column - Is this technology typically used by the patient outside of supervised therapy? [closed]

11.8.d. Other - please specify in the last column - Please tell us any further information you feel is relevant [free text]

12. Within a single treatment session on average how many minutes would you typically spend directly undertaking upper limb treatment with a person who has any severity of upper limb deficits after stroke (i.e. “time on task” so not including paperwork, MDT meetings, transporting patient to gym etc.)? [closed]

12.a. Please use this space to tell us anything you feel is relevant to this question. [free text]

13. On average, how many days a week does a typical person who has had a stroke receive supervised therapy for their upper limb? This can be supervised by a therapist, rehab assistant or other appropriately trained health professional. [closed]

13.a. If you selected Other, please specify: [free text]

14. Within a typical treatment session, what percentage of the entire treatment session would you spend on treatments for the upper limb for each of these presentations?

14.1.a. MILD: someone who is able to move the arm and maintain an arm position against gravity for 10 seconds without physical support - % [closed]

14.2.a. MODERATE: someone who has some movement of the arm but cannot maintain an arm position against gravity for 10 seconds without physical support - % [closed]

14.3.a. SEVERE: someone who has no movement of the arm against gravity OR who can only perform some small movements (e.g. shrugging shoulders) - % [closed]

15. Outside of supervised therapy, does a person who has upper limb deficits after stroke participate in other treatments for their upper limb. This could include using technologies, carer supported exercises or self managed exercises/activities. [closed]

15.a. If yes, please tell us who is involved in this (if appropriate) and how often it occurs (e.g. once a week, everyday, three times a day everyday). If you do not know how often it occurs please still tell us about anyone else who is involved. [closed]

16. MILD DEFICITS: Please list the treatment interventions you use most often for a person who has had a stroke and is able to move their arm and maintain an arm position against gravity for 10 seconds without physical support. [free text]

17. Do you routinely ask a people who have MILD arm deficits to undertake activities for their upper limb in addition to therapist led treatment? [closed]

17.a. If Yes, please tell us what these activities might comprise. If No, please use this space to tell us anything you feel is relevant. [free text]

18. MODERATE DEFICITS: Please list the treatment interventions you use most often for a person who has had a stroke and who has some movement of the arm but cannot maintain an arm position against gravity for 10 seconds without physical support. [free text]

19. Do you routinely ask a people who have MODERATE arm deficits to undertake activities for their upper limb in addition to therapist led treatment? [closed]

19.a. If Yes, please tell us what these activities might comprise. If No, please use this space to tell us anything you feel is relevant. [free text]

20. SEVERE DEFICITS: Please list the treatment interventions that you use most often for someone after a stroke who has no movement of the arm against gravity OR who can only perform some small movements (e.g. shrugging shoulders) [free text]

20.a. Do you routinely ask people with SEVERE arm deficits to undertake unsupervised activities for their upper limb in addition to therapist led treatment? [closed]

20.b. If Yes, please tell us what these activities might comprise. If No, please use this space to tell us anything you feel is relevant. [free text]

21. Please use this space below to provide us with any extra information that you think we may find useful. For instance, you may want to tell us about why you use the treatments you use, or why you have chosen not to use some treatments. [free text]

22. 1. How often do you use constraint induced movement therapy (CIMT) of the arm for someone with arm deficits after stroke? [Likert]

23. If you never use this treatment, please indicate why from the reasons below. [closed]

24. If you selected Other, please specify: [free text]

25. 2. How often do you use any form of electrical stimulation for someone with arm deficits after stroke? [Likert]

26. If you never use this treatment, please indicate why from the reasons below. [closed]

27. If you selected Other, please specify: [free text]

28. How often do you handle/facilitate the arm for someone with arm deficits after stroke? [Likert]

29. If you never use this treatment, please indicate why from the reasons below. [closed]

30. If you selected Other, please specify: [free text]

31. How often do you use functional task practice for the arm for someone with arm deficits after stroke? [Likert]

32. If you never use this treatment, please indicate why from the reasons below. [closed]

33. If you selected Other, please specify: [free text]

34. How often do you use the Graded Repetitive Arm Supplementary Programme (GRASP) for someone with arm deficits after stroke? [Likert]

35. If you never use this treatment, please indicate why from the reasons below. [closed]

36. If you selected Other, please specify: [free text]

37. How often do you use mental practice/mental imagery for someone with arm deficits after stroke? [Likert]

38. If you never use this treatment, please indicate why from the reasons below. [closed]

39. If you selected Other, please specify: [free text]

40. How often do you use mirror therapy for the arm for someone with arm deficits after stroke? [Likert]

41. If you never use this treatment, please indicate why from the reasons below. [closed]

42. If you selected Other, please specify: [free text]

43. How often do you use robot assisted therapy/robotics for the arm for someone with arm deficits after stroke? [Likert]

44. If you never use this treatment, please indicate why from the reasons below. [closed]

45. If you selected Other, please specify: [free text]

46. How often do you use therapy to specifically improve sensation in the arm or hand (e.g. the SENSe programme) for someone with arm deficits after stroke? [Likert]

47. If you never use this treatment, please indicate why from the reasons below. [closed]

48. If you selected Other, please specify: [free text]

49. How often do you use strength training for someone with arm deficits after stroke? [Likert]

50. If you never use this treatment, please indicate why from the reasons below. [closed]

51. If you selected Other, please specify: [free text]

52. How often do you use video gaming or virtual reality training for someone with arm deficits after stroke? [Likert]

53. If you never use this treatment, please indicate why from the reasons below. [closed]

54. If you selected Other, please specify: [free text]

55. Please use this space to tell us about any other treatments that you use and how often you use them. [free text]

56. Did you complete the previous SUPPLES survey (in 2018)? [closed]

57. Please use this space to tell us anything else you think is relevant. [free text]
