# Supplementary material for: Contemporary, postpandemic description of UK occupational therapy and physiotherapy practice to rehabilitate the upper limb after stroke: the SUPPLES 2 online survey
Source: BMJ Open. 2025 Sep 21;15(9):e095290. doi: 10.1136/bmjopen-2024-095290 (PMC12458861; doi:10.1136/bmjopen-2024-095290)
Supplement: online supplemental file 2 [file bmjopen-15-9-s002.docx]

Supplementary File 2

Codes

| **Code** | **Includes** |
| --- | --- |
| Graded Repetitive Arm Supplementary Program | GRASP |
| Repetitive task training (including functional tasks) | Functional training |
|  | Task specific training |
|  | Repetitive task practice |
|  | Task practice |
|  | Repeated movements |
|  | ADL (daily activities) |
|  | Daily tasks |
|  | Tying shoe laces |
|  | Pouring |
|  | Writing |
| Positioning | Stabilising |
|  | Balance |
|  | Corework |
|  | Positioning |
|  | Posture |
| Strength/exercises | Strengthening |
|  | Exercises |
|  | Excs/Ex prog |
|  | Theraband |
|  | Weights |
|  | Manual resistance |
|  | active assisted movements |
|  | resisted |
| Dexterity | Theraputty |
|  | Fine motor |
|  | Picking up small objects |
|  | Dexterity exercises |
| CIMT | CIMT |
|  | constraint |
| Mirror therapy | Mirror exercises |
| Technology | Gripable (specialist eqt) |
|  | Saebo (specialist eqt) |
|  | Motomed (specialist eqt) |
| Electrical stimulation | Electrical stimulation |
|  | NMES (electrical stimulation |
|  | FES |
| Computer games | Computer games |
| Mental practice | Mental practice/imagery |
| Hydrotherapy | Hydrotherapy |
| Education | Education |
| Stretches/ROM | ROM |
|  | stretches |
| Reach and grasp ? functional | Reach and grasp-RTP/functional |
| Splinting | Splinting |
| Sensory re-education | Sensory education |
| Facilitated movements | Facilitated movements |
|  | Guided practice |
| Taping | Taping |
| Weight-bearing | Weight-bearing |
| Oedema management | Oedema management |
| Tone management | Tone management |
| Pain management | Pain management |
| Attention on arm | Attention on arm |
| Botox | Botox |
| Shoulder management | Shoulder management |
| Bimanual activities/training | Bimanual activities/training |
| Mobilisation and massage | Mobilisation and massage |
| Home education programme | Home education programme |
| Apps | Apps |
