# Supplementary material for: Contemporary, postpandemic description of UK occupational therapy and physiotherapy practice to rehabilitate the upper limb after stroke: the SUPPLES 2 online survey
Source: BMJ Open. 2025 Sep 21;15(9):e095290. doi: 10.1136/bmjopen-2024-095290 (PMC12458861; doi:10.1136/bmjopen-2024-095290)
Supplement: online supplemental file 3 [file bmjopen-15-9-s003.docx]

Supplementary File 3

Details of regularity of use for individual interventions

| Intervention | Never | Rarely | Sometimes | Often | Always |
| --- | --- | --- | --- | --- | --- |
| Functional task practice (n=121) | 0 | 0 | 3 | 45 | 73 |
| Handling/facilitation (n=121) | 0 | 2 | 6 | 51 | 62 |
| Strength training (n=122) | 2 | 6 | 19 | 59 | 36 |
| Graded Repetitive Arm Supplementary Program (n=121) | 14 | 8 | 27 | 58 | 14 |
| Sensation training (n=111) | 22 | 14 | 35 | 29 | 11 |
| Mental practice/imagery (n=122) | 9 | 24 | 40 | 40 | 9 |
| Electrical stimulation (n=113) | 34 | 22 | 33 | 21 | 3 |
| Constraint induced movement therapy (n=122) | 36 | 42 | 36 | 6 | 2 |
| Mirror therapy (n=122) | 28 | 34 | 50 | 10 | 0 |
| Video gaming/virtual reality (n=122) | 66 | 35 | 20 | 1 | 0 |
| Robotic assisted therapy (n=122) | 111 | 7 | 1 | 3 | 0 |
