# Supplementary material for: Contemporary, postpandemic description of UK occupational therapy and physiotherapy practice to rehabilitate the upper limb after stroke: the SUPPLES 2 online survey
Source: BMJ Open. 2025 Sep 21;15(9):e095290. doi: 10.1136/bmjopen-2024-095290 (PMC12458861; doi:10.1136/bmjopen-2024-095290)
Supplement: Supplementary file 4 [file bmjopen-2024-095290.reviewer_comments.pdf]

PEER REVIEW HISTORY

BMJ Open publishes all reviews undertaken for accepted manuscripts. Reviewers are asked to complete a checklist review form and are provided with free text boxes to elaborate on their assessment. These free text comments are reproduced below.

ARTICLE DETAILS

Title (Provisional)

A contemporary, post-pandemic description of UK occupational therapy and physiotherapy practice to rehabilitate the upper limb after stroke: The SUPPLES 2 online survey

Authors

Jarvis, Kathryn Anne; Connell, Louise; Peel, Rosemary; Stockley, Rachel C

VERSION 1 - REVIEW

**Reviewer**	**1**
**Name**	**Kolmos, Mia**
**Affiliation**	**Copenhagen University Hospital**
**Date**	**14-Mar-2025**
**COI**	**None**

Abstract:

Design needs to be described more in detail and in a more concise language. Rather unclear how the survey was done. Please use the headlines: background and objectives, methods, results, conclusions. No. of therapists completing the survey should be reported under "results". which therapists the survey were aimed for should be described in methods. Needs 1-2 sentences regarding "background" of the study.

Background:

Robotics and VR can hardly be seen as evidence-based therapeutical interventions. And is definetely not accessible to all.

Conclusion should be written more concisely. "treating the upper limb has not increased compared to 2018" - why should it be increased? Should be stated more neutrally. The direction of therapy amount was not main hypothesis.

Methods

Please write "Methods" in plural in the subheading, not Method.

"An additional question was included about the use of interventions to address sensation to ensure the survey was comprehensive in seeking information about evidence-based interventions" I dont understand this sentence, please rephrase.

Recruitment: How did the authors try do reduce population bias among the respondents? Surely there may be a bias in who will answer the survey in mails and social media. There needs to be a short description of how the recruitment strategy was selected and who was the target of the survey. The authors report working places. What about amount of work experience, e.g., how many years of experience as therapist - was that recorded?

Statistics: The section regarding statistical considerations and analysis is completely missing? The authors only report how data will be presented (as median, IQR etc) and that they were done in excel and spss - but not which analyses and why.

Results:

The tables could be combined to one table i stead of 3 separate tables pr. severity

Table 4 would be more illustrative as a bar chart in stead of a table. Very difficult to get an overview of data.

Table 6: Could be described in text - not helpful.

Duration: How was the time estimated - I expect this to very highly prone to bias, it may be very difficult to estimate expecially retrospectively.

It would be relevant with a section regarding "how much" i.e., the intensity such as number of repetitions per session.

The authors report frequencies of replies but no analyses based on these frequencies such as comparison from 2018 or between employment organisation etc.

Discussion:

In general too long and the purpose of some of the paragraphs is not really clear.

"...a picture of post-pandemic therapeutic practice for the upper limb following stroke" - with so few respondents the results is hardly a picture of the post-pandemic therapeutic practise following stroke.

It is not clear if the aim of the study was to compare the practise reported to the current guidelines or if the aim was to report amount of therapy and method (face-to-face/virtual).

"Previous studies have identified barriers to the adoption of technology, including cost, ease of use, knowledge and skills, and accessibility [22-24]. These barriers must be overcome if therapists are to embrace evidence-based technological interventions in rehabilitation."

How should the barriers be overcome? please elaborate or leave out this section which is not providing any valuable points in the discussion.

A simple factor is the availability of technique - robotics and VR are certainly not standard equipment. And other studies have demonstrated that robotics is not superior to standard "in person" therapy - quite the opposite. See: doi: 10.3390/healthcare12171720

Amount of therapy: The study does not provide any information on WHY the amount of therapy did not meet the national guidelines.

Limitations:

". However, in a busy workforce the length of the survey may have impacted the number of therapists who felt they could take time to respond"

If the survey is too long for a larger group of theapists to respond then the tool has failed it's purpose and should be revised to a more appropriate length.

Reviewer **2**

Name **Donoso Brown, Elena V.**

Affiliation **Duquesne University John G Rangos Sr School of Health Sciences**

Date **12-Jun-2025**

COI **I have done similar work in this field but in the United States.**

Background

The background clearly articulates the importance of upper limb rehabilitation post-stroke, the previous survey that was completed and the rationale for the update.

The discussion there is a strong connection or emphasis on the updated guidelines, but these are not clearly addressed or considered as part of the introduction. Recommend a revision to include this possibly in the paragraph that discusses recent changes to the NHS.

While the objective was clearly stated, no specific hypothesis about what would be found were defined, if there were no a priori hypotheses, please state this.

Methods

Pg 3 line 31: It is noted that there were wording changes from the previous survey, please provide at least one example to allow a reader to better understand how substantially these clarifications may have impacted the responses of the participants.

There were 57 total questions of three different types, can you please break down for a reader the number of each type of question.

Also information on the organization of the survey could be more clearly presented in the narrative to provide a reader greater information about the general structure

Could the authors please clarify the type of information provided on the information sheet prior to agreeing to complete the survey? It is suggested that this may be information typical to a consent form but it would be clearer to state what type of information was provided.

Also how many times was it e-mailed out in those 12 weeks? Or any indication/approximation of the number of times it was posted on social media and which channels?

For data analysis - It is recommended to add subheadings to this section and re-organize to address data processing/cleaning (what software was used, how certain responses were label) and then analysis (use of median and ranges). The current organization is somewhat difficult to follow. Also reporting the percent of fully completed surveys and management of missing data in this section should be addressed as well.

There is a need to provide more description about the approach to the open-ended information, as what is presented seems to apply mostly to the content questions, but it is not clear how the listing or grouping was done. Was there a codebook generated that defined what items were grouped together into themes? If so this would be a great addition to the supplement. It would also provide the readers with information on the other interventions used less frequently which given the sizable number of these interventions may be of interest.

Also related to this section, how was 10% selected to represent inconsistency of use in practice? It would seem that one could argue that anything that fewer than 50% of therapists are reporting could be inconsistently used. More information on this choice would be helpful.

While the comparison to the 2018 survey is appreciated and a driver for this paper, I would suggest moving these comparisons to the discussion as this is the most common place for the location of connections to previous work, especially as these data have already been published.

Results

Recommend leading with the overall summary of interventions, what is currently in Table 4 and then moving into the break down by severity.

The section on content also seems to contain information on frequency by severity, consider moving this information to the section on frequency or duration.

In reviewing the survey, participants also had a space to write in why an intervention was not used, but no data on this are presented. Please address this in the paper.

As noted above recommend removing the references to the 2018 survey in the results and addressing these in the discussion.

One question which might be beyond the scope of the paper was where there any difference in recommendations based on either the amount of case load post-stroke or the years of

practice? Given that there was no minimum amount of experience or exposure to clients post-stroke this could increase variability and I wonder if patterns of intervention use were clearer for therapists who saw persons post-stroke more often and possibly were more experienced clinicians.

Discussion

Recommend revising the first paragraph to focus on a brief summary of the results, unless the current format is required by BMJ, as the point about face to face practice and connecting this to the literature can stand on its own in a separate paragraph.

While data were gathered on unsupervised practice/therapy this was not included in the discussion, despite the recognition that treatment time falls below what is needed for upper limb interventions. This also ties directly into the use of technology which was also limited, as technologies have the potential to support unsupervised practice by clients. Recommend addressing these elements in the discussion.

Please review the paragraph on dosage, while I agree that people post-stroke do not receive services at the levels stated in the guidelines, the survey asked only about upper limb interventions and extrapolating how many sessions they receive overall that address other goals, seems to stretch beyond the data gathered. Recommend revising to focus on the limitation specifically to UL rehabilitation and the evidence related to intensity needed to create effective change at each level of severity, as this is mentioned but not referenced in the discussion.

Limitations

Timing of the survey, while the authors attribute this to possible workload issues, the survey was also given at a time of the year when people are typically celebrating various holidays which also could have impacted response rate. It is not clear if the timing was the same for the first survey.

Recommend naming selection bias specifically in that people either more interested, “confident in their practice or adherence to evidence-based guidelines” responded.

Also given the small nature of the sample predominantly from England, more evidence to suggest that this sample is representative is needed to support the conclusion that this survey is a complete reflection of current practice related to upper limb rehabilitation in the UK. For example, is there a workforce survey that mirrors the location of the therapists that has a similar distribution.

VERSION 1 - AUTHOR RESPONSE

Reviewers’ comments	Response
Reviewer 1	
ABSTRACT	The format of the abstract reflects the BMJ submission guidance. We have aimed to

Design needs to be described more in detail and in a more concise language. Rather unclear how the survey was done. Please use the headlines: background and objectives, methods, results, conclusions. No. of therapists completing the survey should be reported under "results". which therapists the survey were aimed for should be described in methods. Needs 1-2 sentences regarding "background" of the study.	address the reviewer's comments whilst maintaining the broad structure of the submission requirements.<br><br>We have moved the no. of therapists completing the survey as suggested and improved conciseness of language. **P.1**
Conclusion should be written more concisely. "treating the upper limb has not increased compared to 2018" - why should it be increased? Should be stated more neutrally. The direction of therapy amount was not main hypothesis.	Thank you for this comment. The sentence has been removed and a small change to the remaining sentences has been made to increase neutrality. **P.1 lines 21-25**
Background:<br>Robotics and VR can hardly be seen as evidence-based therapeutical interventions. And is definitely not accessible to all.	Thank you for this observation. These have been removed from this paragraph **Background P1**
Methods<br>Please write "Methods" in plural in the subheading, not Method.	Changed as requested **P3 line 16**
Methods<br>"An additional question was included about the use of interventions to address sensation to ensure the survey was comprehensive in seeking information about evidence-based interventions" I don't understand this sentence, please rephrase.	Thank you for this observation. This has been re-phased **P. 3 lines 22-26**
Methods<br>Recruitment: How did the authors try do reduce population bias among the respondents? Surely there may be a bias in who will answer the survey in mails and social media. There needs to be a short description of how the recruitment strategy was selected and who was the target of the survey. The authors report working places. What about amount of work experience, e.g., how many years of experience as therapist - was that recorded?	We have added further detail about the recruitment strategy. **P.4 lines19-28**<br>It is inevitable, that there will be respondent bias, with therapists who do not access professional channels or social media for their work being less likely to respond. This has been recognised in the Limitations. **P. 14 lines 25-28**<br>We collected:<br>How many years have you been qualified?<br>What is your highest academic qualification?<br>How many years have you worked with people who have had a stroke?<br>These questions are stated in the **Supplementary File 1**
Methods:<br>Statistics: The section regarding statistical considerations and analysis is completely missing? The authors only report how data will be presented (as median, IQR etc) and	Additional detail about the analysis and considerations have not been added. **P.5 line 18-P.6 line 2**

that they were done in excel and spss - but not which analyses and why.	
Results:<br>The tables could be combined to one table in stead of 3 separate tables pr. severity<br>Table 4 would be more illustrative as a bar chart instead of a table. Very difficult to get an overview of data.	Thank you for this feedback, we recognise that Tables 1-3 could be represented in one table, however, this format is reflective of the style the data was presented in the original paper reporting the 2018 survey, so it is our preference to maintain the three tables if this is acceptable.<br>**Table 4 has been presented as a bar chart (Fig 2)**
Table 6: Could be described in text - not helpful.	This has been added to the text P**. 12 lines 6-11** and the table removed
Duration: How was the time estimated - I expect this to very highly prone to bias, it may be very difficult to estimate especially retrospectively.	This detail has been added **P. 4 lines 3-7**<br>The risk of bias is recognised in the limitations "the responses were self-reported, so it must be recognised that the findings reflect the therapist respondents' perceptions of what they do, rather than the findings being based on observation" **P.14 lines 23-25**
It would be relevant with a section regarding "how much" i.e., the intensity such as number of repetitions per session.	We did not collect data at this granular level as it was out of the scope of the paper.
The authors report frequencies of replies but no analyses based on these frequencies such as comparison from 2018 or between employment organisation etc.	Thank you for this observation, this has now been added as part of the Discussion, which is in line with the second reviewer's comments. **P. 15 lines 4-11**
Discussion:<br>In general too long and the purpose of some of the paragraphs is not really clear.	This section has been reviewed and re-crafted to increase clarity.
"...a picture of post-pandemic therapeutic practice for the upper limb following stroke" - with so few respondents the results is hardly a picture of the post-pandemic therapeutic practise following stroke.	This sentence has been removed
It is not clear if the aim of the study was to compare the practise reported to the current guidelines or if the aim was to report amount of therapy and method (face-to-face/virtual).	The aim was to describe current practice, but we have endeavoured to consider both these components as part of our analysis. We have altered the paragraphs to reflect our ambition. **P.12 line 16-P.13 line 6**
"Previous studies have identified barriers to the adoption of technology, including cost, ease of use, knowledge and skills, and accessibility [22-24]. These barriers must be overcome if therapists are to embrace evidence-based technological interventions in rehabilitation."<br>How should the barriers be overcome? please elaborate or leave out this section	Sentence removed

which is not providing any valuable points in the discussion.	
A simple factor is the availability of technique - robotics and VR are certainly not standard equipment. And other studies have demonstrated that robotics is not superior to standard "in person" therapy - quite the opposite. See: doi: 10.3390/healthcare12171720	We agree that effectiveness and accessibility are likely to limit use of some interventions. We have added further clarification **P.7 lines 27-29.**
Amount of therapy: The study does not provide any information on WHY the amount of therapy did not meet the national guidelines.	This is not within the scope of this study, but we agree this work is important.
Limitations:<br>". However, in a busy workforce the length of the survey may have impacted the number of therapists who felt they could take time to respond"<br>If the survey is too long for a larger group of therapists to respond then the tool has failed it's purpose and should be revised to a more appropriate length.	Thank you for this interesting reflection. To meet its purpose, the survey did need to be detailed. However, we do feel that the time required to complete may have reduced the number of people who responded. Those who did respond completed a full or almost full data set. We therefore gained a detailed and comprehensive overview of 122 therapists' practice, and we have been able to describe contemporary, post-pandemic occupational and physiotherapy practice to rehabilitate the upper limb after stroke.
Reviewer 2	
Introduction:<br>The background clearly articulates the importance of upper limb rehabilitation post-stroke, the previous survey that was completed and the rationale for the update.	Thank you
The discussion there is a strong connection or emphasis on the updated guidelines, but these are not clearly addressed or considered as part of the introduction. Recommend a revision to include this possibly in the paragraph that discusses recent changes to the NHS.	Some sentences have been added to strengthen the content that relates to the updated guidance in the first paragraph. **P. 2 lines 14-16**
While the objective was clearly stated, no specific hypothesis about what would be found were defined, if there were no a priori hypotheses, please state this.	This has been added **P. 3 line 13-14**
Methods:<br>Pg 3 line 31: It is noted that there were wording changes from the previous survey, please provide at least one example to allow a reader to better understand how substantially these clarifications may have impacted the responses of the participants.	Example added **P. 3 lines 27-30**
There were 57 total questions of three different types, can you please break down	The question types are complicated to summarise as some of the questions have

for a reader the number of each type of question.<br>Also information on the organization of the survey could be more clearly presented in the narrative to provide a reader greater information about the general structure	multiple parts. We have therefore placed this information in the **Supplementary File 1** along with the questions as we feel this is a more comprehensive summary.<br>Changes have been made to this section to increase clarity.
Could the authors please clarify the type of information provided on the information sheet prior to agreeing to complete the survey? It is suggested that this may be information typical to a consent form but it would be clearer to state what type of information was provided.	Information added **P.4 lines 20-24**
Also, how many times was it e-mailed out in those 12 weeks? Or any indication/approximation of the number of times it was posted on social media and which channels?	This detail has been added to **P.4 lines 29-31.**
For data analysis - It is recommended to add subheadings to this section and re-organize to address data processing/cleaning (what software was used, how certain responses were label) and then analysis (use of median and ranges). The current organization is somewhat difficult to follow. Also reporting the percent of fully completed surveys and management of missing data in this section should be addressed as well.	This section has been re-organised as suggested. Further clarification has been added, including details about number of datasets included and how missing data was managed. **P5 line 5 – P.6 line 2**
There is a need to provide more description about the approach to the open-ended information, as what is presented seems to apply mostly to the content questions, but it is not clear how the listing or grouping was done. Was there a codebook generated that defined what items were grouped together into themes? If so this would be a great addition to the supplement. It would also provide the readers with information on the other interventions used less frequently which given the sizable number of these interventions may be of interest.	The main free-text responses did relate to the content of interventions and the unsupervised activities. The other free-text responses were only used where respondents wanted/needed to add clarification.<br>Given the discrete nature of the data we developed code list rather than a traditional code book. This has been added as **Supplementary File 3**. Further detail has been added to the text **P.5 lines 24-29**
Also related to this section, how was 10% selected to represent inconsistency of use in practice? It would seem that one could argue that anything that fewer than 50% of therapists are reporting could be inconsistently used. More information on this choice would be helpful.	Thank you for this comment. The aim was to remove interventions that were rarely used by therapists. The wording has been altered to reflect this. **P.5 line 27-29**
Results<br>While the comparison to the 2018 survey is appreciated and a driver for this paper, I	The 2018 survey results have been removed to the Discussion section. We have left the detail about the 2018 results in Tables 3-5, as

would suggest moving these comparisons to the discussion as this is the most common place for the location of connections to previous work, especially as these data have already been published.	we feel this provides valuable context for the 2023 results.
Results<br>Recommend leading with the overall summary of interventions, what is currently in Table 4 and then moving into the break down by severity.	Table 4 has been changed to a bar chart (**Fig 2**) on recommendation of another reviewer and moved to the start of the Content section.
The section on content also seems to contain information on frequency by severity, consider moving this information to the section on frequency or duration.	We have spend some time considering the placement of this information and, as the data relates to the proportion of the session that is focused on the upper limb, and we feel this is best placed within the section on content of the session.
In reviewing the survey, participants also had a space to write in why an intervention was not used, but no data on this are presented. Please address this in the paper.	Thank you for this helpful comment. We have now added this information **P. 7 lines 27-31**
One question which might be beyond the scope of the paper was where there any difference in recommendations based on either the amount of case load post-stroke or the years of practice? Given that there was no minimum amount of experience or exposure to clients post-stroke this could increase variability and I wonder if patterns of intervention use were clearer for therapists who saw persons post-stroke more often and possibly were more experienced clinicians.	This is beyond the scope of this paper, but we agree would be worthy of further investigation.
Discussion:<br>Recommend revising the first paragraph to focus on a brief summary of the results, unless the current format is required by BMJ, as the point about face to face practice and connecting this to the literature can stand on its own in a separate paragraph.	Thank you, a brief summary of the results has been added (**Discussion, 1st paragraph**) and the paragraph about face-to-face practice is now a stand alone paragraph **P.12 lines 31-P.13 line 6**
While data were gathered on unsupervised practice/therapy this was not included in the discussion, despite the recognition that treatment time falls below what is needed for upper limb interventions. This also ties directly into the use of technology which was also limited, as technologies have the potential to support unsupervised practice by clients. Recommend addressing these elements in the discussion.	Thank you, this has now been included. **P. 13 lines 18-26**
Please review the paragraph on dosage, while I agree that people post-stroke do not receive services at the levels stated in the guidelines,	We have changed the focus of this paragraph to place the focus on intensity. **P.14 lines 10-18**

the survey asked only about upper limb interventions and extrapolating how many sessions they receive overall that address other goals, seems to stretch beyond the data gathered. Recommend revising to focus on the limitation specifically to UL rehabilitation and the evidence related to intensity needed to create effective change at each level of severity, as this is mentioned but not referenced in the discussion.	
Limitations<br>Timing of the survey, while the authors attribute this to possible workload issues, the survey was also given at a time of the year when people are typically celebrating various holidays which also could have impacted response rate. It is not clear if the timing was the same for the first survey.	We recognise that whilst the survey was open over a traditionally festive time, it was open for 3 months, therefore negating some of this concern, therefore we did not include in the limitations for the study.
Recommend naming selection bias specifically in that people either more interested, "confident in their practice or adherence to evidence-based guidelines" responded.	We have added this additional detail, thank you **P.14 lines 25-28**
Also given the small nature of the sample predominantly from England, more evidence to suggest that this sample is representative is needed to support the conclusion that this survey is a complete reflection of current practice related to upper limb rehabilitation in the UK. For example, is there a workforce survey that mirrors the location of the therapists that has a similar distribution.	Further detail added to demonstrate alignment between numbers recruited in each of the UK home nations and the workforce **P.15 lines 4-12**
